# Perspective: Optimizing Host Environments to Enhance MSK Tissue Repair and Complement Use of Cellular Therapies Is Critical for Improving Outcome Success—An Unmet Need

**DOI:** 10.3390/cells15151317

**Published:** 2026-07-23

**Authors:** David A. Hart

**Affiliations:** Department of Surgery, Faculty of Kinesiology, and McCaig Institute for Bone & Joint Health, University of Calgary, Calgary, AB T2N 4N1, Canada; hartd@ucalgary.ca

**Keywords:** cell therapy, tissue engineering, biomaterials, in vivo environment, controlling inflammation, optimizing implantation sites

## Abstract

**Highlights:**

**What are the main findings?**

- Considerable research effort over the past >35 years with MSC, iPSC and EV to improve the repair of MSK tissues has met with limited and often variable in vivo success.
- In contrast, optimizing the host environment via controlling inflammation, modifying tissues altered by chronic injury or disease, as well as host variables (i.e., age, sex, location, genetics, co-morbidities) has not received coordinated attention.

**What are the implications of the main findings?**

- This Perspective article discusses the needs regarding optimizing host variables and some possible approaches that may lead to achieving enhanced repair/regeneration.
- The integration of excellent cell-scaffold interventions with an optimized injury site environment will lead to improved protocols for the successful repair of damaged MSK tissues.

**Abstract:**

Since the discovery and characterization of mesenchymal stem cells (MSC) some 40 years ago and the development of induced pluripotent stem cells (iPSC), opportunities to advance repair and potentially regenerate injured or diseased connective tissues of the musculoskeletal (MSK) system have been intensely investigated. While some advances have been made with some tissues, progress has not been as great as was hoped following the availability of such stem-like cells. Optimizing cell-biomaterial composites in vitro for features such as cell state or extracellular vesicle cargo, structure, composition, biomechanical integrity are likely to be only part of the story and optimizing the environments where such composites have been implanted has not received the same attention. Thus, implanting optimized or near optimized cell-biomaterial composites in acutely or chronically affected implantation sites can compromise the efficacy of such composites to effectively repair/regenerate MSK tissues. This may be due to inflammatory processes, alterations to the remaining tissues in the injury site during development of chronicity, waiting too long to repair residual tissue, or other host factors. Thus, success in realizing the potential of MSC and iPSC may depend, in significant part, on the environment at the implantation site. Further attention to optimizing the implantation site may enhance outcome success with long-term return to tissue function. Furthermore, heterogeneity in patient populations regarding natural factors influencing healing outcomes after injury may also require changes to clinical trial design to assess efficacy of cell therapies.

## 1. Introduction

### 1.1. Purpose

Over the past >35 years, many studies have attempted to repair the connective tissues of the musculoskeletal system (MSK system). These tissues serve a variety of functions in very mechanically active environments and in the individual tissues (i.e., articular cartilage, ligaments, tendons, menisci). In adults, these tissues are hypocellular with several of them exhibiting a relatively low ability to undergo endogenous effective repair once injured or compromised by disease. Being in biomechanically active environments of varying intensities also means that most of the tissues are very hypovascular and hypoinnervated. In addition, such environments may not be able to effectively initiate and develop a healing response leading to function outcomes.

A large number of studies with a variety of somatic or stem/stromal cells +/− biomaterials have been undertaken in both preclinical models and in human populations attempting to facilitate exogenous repair of these compromised MSK tissues. These efforts have met with limited success in many of the studies, even when so-called mesenchymal stem cells (MSC) with different relevant lineage capability were used. While many studies have attempted to optimize the in vitro biological and biomechanical features of the cells and biomaterials used, widespread success has been limited and challenging.

This perspective article first presents an outline of the approaches that have been used to enhance repair and some of their limitations. It then outlines the need to better understand the requirement to characterize and improve the host environment to achieve further success in repairing injured or diseased tissues of the MSK system, and then ends with an outline of possible direction to enhance the translation potential of the findings. Thus, the environment where cells and tissue engineered composites are implanted in vivo are likely abnormal and not optimized for the acceptance of the implants, particularly if the in vivo condition has transitioned to a chronic state. Without improving such host environments will contribute to only limited or variable success, or failure. Thus, both optimized implants with an appropriate environment to place them into are required for long-term effective repair of these MSK system tissues.

### 1.2. Background

#### 1.2.1. MSK Tissue Growth and Development

**Figure 1:**
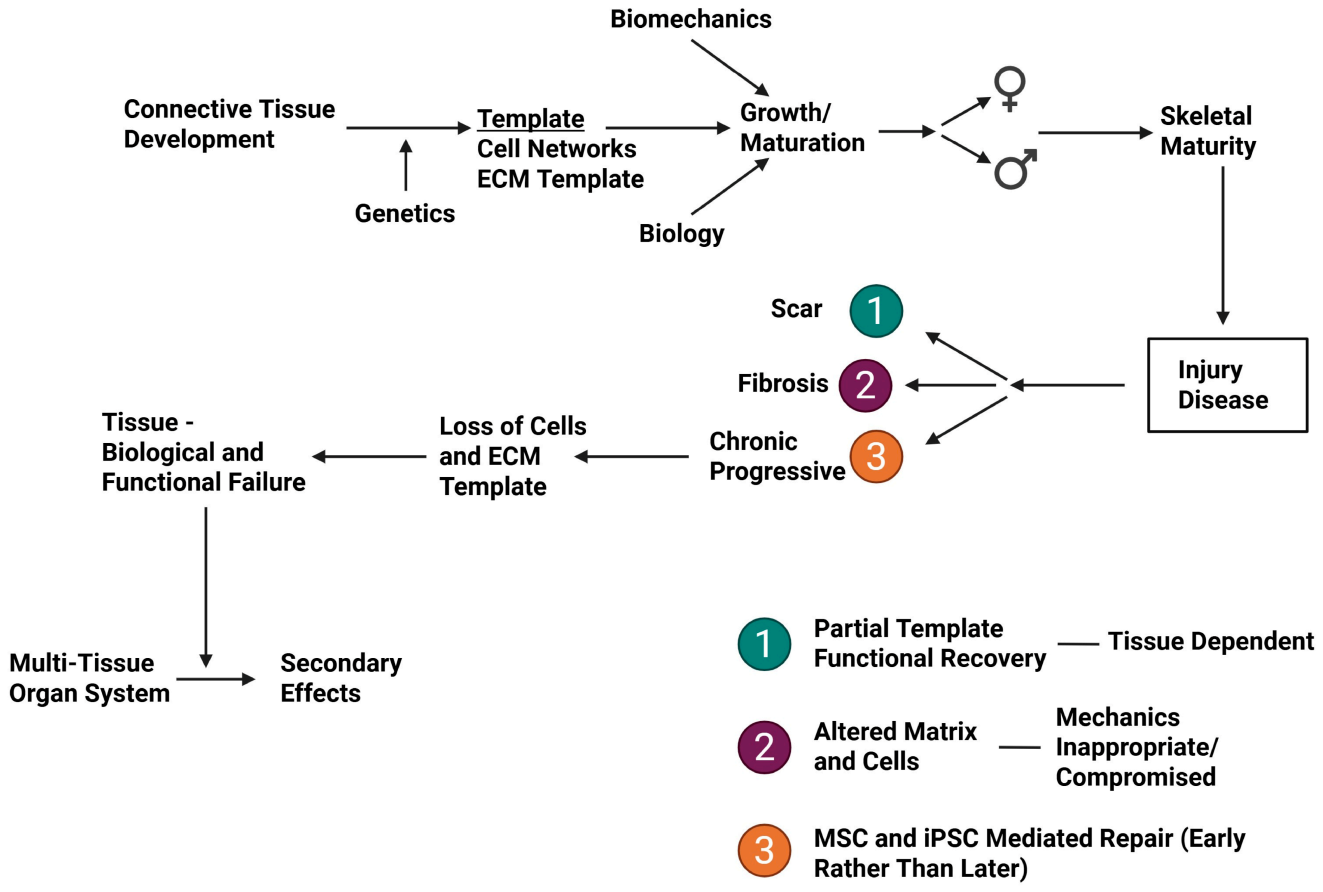
Outline of complexity of connective tissue growth, maturation and responses to injury and disease. Following injury or disease development, the tissue may develop scar tissue which recovers some function in a tissue-dependent manner, develop a fibrotic response with deposition of an amorphous collagen-rich ECM which is non-functional, or the condition may become chronic and in need of exogenous interventions with MSC or iPSC containing composites to foster repair and recovery of some function.

At the time of birth, connective tissues of the MSK system such as ligaments and others are “cell-rich and matrix poor” [1,2,3]. However, the extracellular matrix (ECM) develops as a template for future growth and maturation as the biomechanical demands increase [4], and this is outlined in Figure 1. Some of these templates are quite complex [5], making it difficult to functionally recapitulate once injured. Growth and maturation continue into puberty with the tissues becoming more ECM-rich as the cells become farther and farther apart. However, the cells on connective tissues such ligaments and tendons appear to remain in contact via gap junctions [6,7], as well as in parts of the annulus fibrosus [8]. In addition, cells in the outer, ligament-like part of the meniscus are connected, while those in the inner compressive environment appear to exist as single cells [9]. For tissues of a joint such as the knee, there has to be coordinated growth of the various tissues to ensure proper functioning, a situation that has led to consideration of such joints as organ systems [10,11,12].

With the onset of puberty, sex-specific responses to the changing hormonal environment leads to hormonal influences on the connective tissues of the MSK system [13,14,15]. However, it should be noted that the joints of all females do not respond similarly to hormonal variations [15], and thus there is heterogeneity in the female population. Thus, even during exposure to similar alterations in sex hormone levels, some females experience increases in joint laxity while others do not [16]. However, the dynamic nature of regulation of joint function in some females is reported to increase risk for sport-related joint injuries [17] such as anterior cruciate ligament ruptures (ACL) [18,19].

Additional sex-specific influences on connective tissues and their function occur in females via pregnancy and lactation [20]. Many also experience loss of bone calcium to support fetal growth and the neonate via milk production [21,22,23]. For many females, the bone can recover following weaning [24,25]. However, for others that develop MSK conditions during pregnancy, such as those developing low back pain, the pain does not subside post-partum and may get worse with subsequent pregnancies, leading to increased risk for development of intervertebral disc disease later in life [26,27].

The onset of menopause in females also can lead to increased risk of loss of joint integrity and increased risk for development of degenerative joint diseases such as osteoarthritis (OA) in a subset of such females [28]. Before menopause, the incidence of OA in females and males is ~1/1 but after menopause the incidence in females versus males is >2/1 [17,28,29]. Thus, loss of sex hormones in such females contributes to loss of some regulatory control of joints in these females.

Given the above discussion, it is likely that the effective repair of compromised connective tissues will have to address several age- and sex-specific considerations. Whether such considerations are similarly important in all connective tissues of the MSK system remains to be defined.

#### 1.2.2. Biomechanical Environments

Different connective tissues of the MSK system function in different mechanical environments [30]. This is reflected in the composition and organization of the tissue [4,31,32]. Thus, the ACL consists of two bands of tissue and functions in a high load environment in the interior of the knee. In contrast, the medial collateral ligament (MCL) is a stabilizing ligament external to the knee and operates in a low load environment. Similarly, the Achilles tendon (AT) operates in a high load environment while the supraspinatus tendon (SST) of the shoulder operates in a lower mechanical environment and has a unique structure and composition [33,34]. The menisci of the knee are also complex as the inner part of the tissue is subjected to compressive loads while the outer part of the tissue is more like a ligament that resists the compressive loads applied to the inner part of the tissue (discussed in [4]). Some parts of the knee menisci are also innervated and vascularized [35,36], factors which could have influence on repair potential. Similarly, the nucleus pulposus (NP) of the intervertebral disc (IVD) functions in a compressive loading environment and the outer annulus fibrosus (AF) has a complex structure that resists the compression of the NP, and with the bone and endplates the IVD functions as an integrated organ system [37]. Thus, the repair potential for these components may vary considerably.

Finally, articular cartilage operates in complex loading environment, and the tissue is subjected to both shear and compression loading, and the properties exhibit heterogeneity in location (discussed in [38,39,40,41]). Further, the loading environment is heterogeneous, with some areas being loaded more in normal walking and running than other areas. This is reflected by heterogeneity in properties, what the cells are making, and how they are organized in different zones. Thus, the surface zone cells are flattened and uniquely make boundary lubricants such as PRG4 [42,43,44], and the middle zone cells make a number of proteoglycans, and the cells are rounded and organized in columns [45]. These tissues are effectively aneural and avascular and thus have very limited endogenous repair potential.

Thus, the composition and complexity of the ECM of these tissues which function in diverse mechanical environments present serious challenges to their effective exogenous repair with a return to function once compromised. Such challenges lie in both the cells involved, as well as the composition and organization of the repair tissue, both of which would require recapitulation of developmental programs for regeneration. The integration of mechanical and biological factors into a complex ECM template presents challenges for effective functional repair [46].

#### 1.2.3. Genetic Influences on Connective Tissues

The above discussion regarding composition of various connective tissues was somewhat generalized to the majority of the population. There may be some variation in composition based on factors such as genetic influences. For example, connective tissues of individuals with Marfan’s syndrome, Ehlers–Danlos syndromes or Loeys–Dietz syndrome can have mutations in molecules such as collagens that are common to such tissues (reviewed in [47,48]). Subsets of some of these syndromes can also have mutations in growth factors, growth factor receptors and other molecules that could impact the composition and potentially the organization of the tissues [47]. Such genetic alterations can influence the function of the tissues, such as range of motion for joints, possibly the laxity of joints, and the response to hormones [16]. The presence of such mutations often increases the risk for injury of tissues [49,50,51] and compromised healing once injured [52]. If one was considering enhanced repair with exogenous cells and biomaterials in these populations, one has to face the issue of using autologous cells or allogeneic cells, variables that would impact the outcomes.

#### 1.2.4. Loss of MSK Tissue Integrity (Injury and Disease)

**Figure 2:**
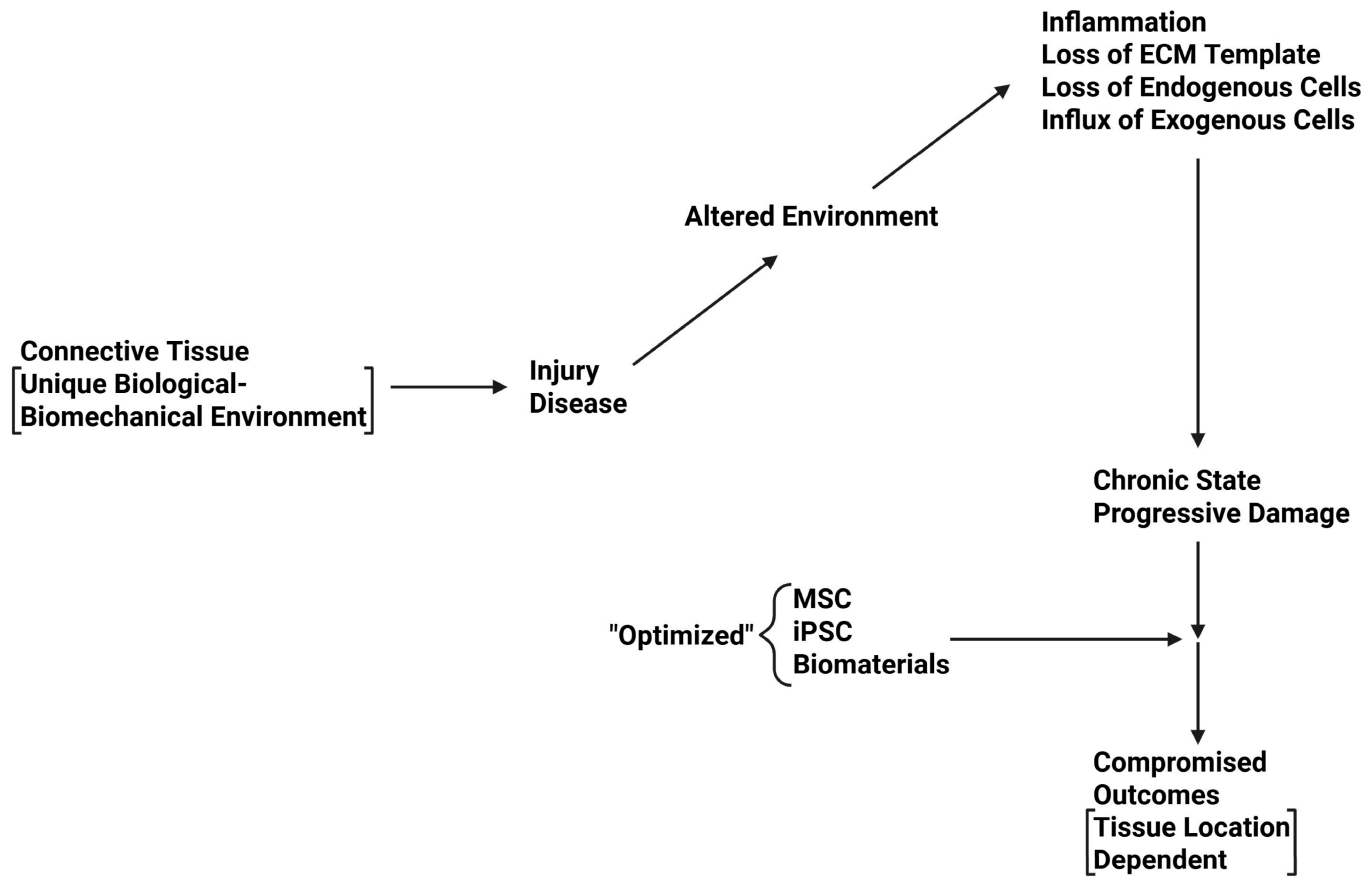
Outline of the response of a connective tissue to injury/disease, leading to an adapted environment which contributes to further tissue damage. Such environments may resist the reparative potential of optimized MSC or iPSC biomaterial composites.

Connective tissues of the MSK system can become altered and lose their integrity due to a number of reasons, many of which affect their ability to initially repair. Such factors include aging, development of comorbidities such as diabetes [53,54,55] or cardiovascular conditions [56], responses to drugs [57], development of overuse syndromes [58,59], overt injury and trauma, development of obesity and its consequences [60,61], or environmental variables (i.e., smoking, excessive alcohol use, poor nutrition). Some of these are chronic conditions that can progress to tissue failure (i.e., tendinosis to tendon rupture), some are acute injuries that can progress to a chronic degenerative condition (i.e., cartilage defect from trauma evolving to osteoarthritis), some can secondarily impact the healing process (i.e., diabetes, cardiovascular disease, smoking, metabolic conditions). These variables and their heterogeneity, as well as unique biomechanical environments [30] present significant challenges to developing effective interventions to enhance tissue repair (Figure 2).

It should also be noted that there are neuroregulatory influences on wound healing in connective tissues. This has been demonstrated for skin [62,63], tendons [64], and likely other innervated connective tissues. Thus, patients with peripheral neuropathy may not only have compromised endogenous tissue repair, but also exogenous tissue repair.

## 2. Autologous and Facilitated Repair

Autologous repair of an injured connective tissue of the MSK system may progress through a series of wound healing steps leading to return to at least partial function for some tissues (i.e., ligaments, tendons, skin, bone), but for other tissues such as the ruptured ACL or damaged articular cartilage, autologous repair does not occur. Therefore, for some damaged tissues, repair may be facilitated by non-cellular approaches or cellular therapies using stem-like cells or endogenous cells (i.e., chondrocytes).

The most direct approach to improving repair of connective tissues of the MSK system would be to attempt to enhance the ability of autologous cells to facilitate effective repair. Thus, one could supplement the wound environment with anabolic growth factors [65], anti-sense interventions [66], platelet-rich plasma [67,68,69] or molecules to enhance autologous cells or negate some impediment to healing [70]. In the latter situation, the use of glucocorticoids has been widespread [71,72,73,74,75]. However, high dose steroids should be avoided as such doses can impact MSC function [76].

The real challenge regarding enhancing healing in this manner, due to either intrinsic or stochastic factors, is that outcomes can be heterogenous, and one does not know which are destined to yield a good outcome or a poor outcome. If one waits until it is very evident, then a “window of opportunity” may have been passed and thus, lost. This has led to investigators trying to identify early biomarkers of good versus poor outcomes (reviewed in [77]). While a promising field, it is still emerging, particularly using proteomic approaches to identify relevant biomarker molecules. The challenges include which fluid or tissue to use as a starting material, the timing of the assessment, and heterogeneity in early responses to an injury.

Finally, because of their location some connective tissue injuries heal well and others do not. For example, injuries to the MCL heal well in their environment outside the knee and in close proximity to the joint capsule while rupture of the intraarticular ACL results in retraction of the torn ends and there is no mechanism for the two torn ends to- find each other. Similarly, a bucket-handle tear of the meniscus leads to the displacement of the torn segment and under loading conditions cannot effectively repair itself. Attempts to fix the torn segment to the remainder of the meniscus leads to frequent re-injury [78]. Similar considerations occur with tears of the SST [79] and in injuries to articular cartilage leading to generation of articular defects or osteochondral defects. These scenarios also pose challenges to effective exogenous repair.

### 2.1. Mature Chondrocytes and Cartilage Repair

For several years, cartilage tissues in the periphery of joints were harvested, differentiated chondrocytes isolated and expanded, and then implanted into areas of the articular cartilage damaged by injury or OA (reviewed in [80,81,82]). The cells were implanted in a defect and then covered with a thin covering that was sutured into place. The outcomes were mixed, with some patients having good outcomes with repair tissue forming and regaining partial integrity. However, this approach was limited in many respects, resulting in damage to other parts of the tissue in order to obtain chondrocytes, a process that can result in joint inflammation, and the cells were placed in the damaged tissue without modifying the implant sites. Furthermore, the isolated autologous cells were often from elderly individuals so they may have been compromised by the age of the affected individual. While the isolated cells were chondrocytes, they had no template to rebuild an ECM that could function in the specific mechanical environment. Thus, this approach had several limitations [83], such as those related to cell source, being implanted without a residual natural template to mimic, and being implanted into an altered biomechanical environment. The approach also required a covering that needed to be sewed into the margins of the tissue to contain the cells. Thus, while used for >20 years, the approach of employing autologous chondrocytes to repair damaged cartilage has led to modest long-term outcomes.

### 2.2. Mesenchymal Stem/Stromal Cells

In the late 1980s–early 1990, Caplan’s group reported on the isolation of cells, initially labeled mesenchymal stem cells (reviewed in [84,85]) (sometimes also called mesenchymal stromal cells), cells that could be differentiated to different lineages in vitro, including chondrocytes, bone cells and adipocytes [86] with differentiation influenced by aging [87]. Such cells could be obtained from bone marrow, synovial membranes, adipose tissue, Wharton’s jelly, placenta, and other sources (reviewed in [88,89]). MSC from specific sources appeared to have some selectivity towards some lineages [90]. For example, those from synovial membranes or synovial fluid preferred chondrogenesis [90].

One of the potential limitations of using autologous MSC for repair of MSK tissues is that they can be influenced by the in vivo inflammatory state from which they were derived [91]. Furthermore, expansion of MSC in vitro can lead to alterations in their characteristics [92]. Therefore, using well characterized allogenic MSC could lead to a more standardized cell source and reproducible outcomes. Such cells are reported to exhibit low immunogenicity (discussed in [93,94]), so immune rejection should not be an issue. The safety of both autologous and allogeneic MSC has been investigated in numerous studies (reviewed in [95]), and they have been found to be safe.

The actual role of cells that are called MSC (stem or stromal) in vivo during the lifespan is not known in detail and additional roles beyond tissue repair have been suggested [96,97]. In these other roles, MSCs may secrete or release factors or vesicles that could influence their paracrine environment. Such possibilities led Caplan [98] to suggest the name for these cells be changed to Medicinal Signaling Cells which would reflect the fact that such cells may function primarily to supply needed signals to other cells. This concept has also been postulated by others [97] and supported by studies with extracellular vesicles (EV, discussed below).

### 2.3. Induced Pluripotent Stem Cells (iPSC)

While MSC can be obtained from a variety of tissue sources, obtaining autologous MSC requires invasion to obtain the starting material. Also, the numbers and perhaps their “quality” decline with age, a stage in life that such cells are often needed (discussed in [97]). An alternative to MSC is the use of induced pluripotent stem cells (iPSC) which can be generated from somatic cells such as skin cells via use of specific reagents developed by Yamanaka’s group ([99,100]; reviewed in [101]). Early protocols used Myc as one of the factors, but this was eliminated due to its tumorigenic potential [102]. A number of studies have examined the potential of iPSC-based interventions for the repair of articular cartilage [103,104,105,106] and other connective tissues of the MSK system [107,108]. Thus, these modified somatic cells offer potential for use in tissue engineering efforts to repair damaged MSK tissues.

### 2.4. Extracellular Vesicles (EV)

In vivo studies such as that of Nakagawa et al. [108] that used human tissue-engineered constructs to repair osteochondral defects in nude rats found that the defects were repaired but no human cells were detected in the mature repair tissue, only rat cells. This type of study has led to the concept that the implanted cells may not be retained in the repair tissue and that they may only stimulate endogenous cells to facilitate the repair. They could do this via secreting anabolic factors including release of extracellular vesicles containing a cargo of anabolic molecules including growth factors, miRNA, proteins, and lipids [109,110,111,112,113]. While widely studied, EV are difficult to standardize as they can vary by donor age, sex, and cell source, as well as preparation method [114].

In spite of limitations, EVs have been investigated and proposed for use in repairing a variety of tissues [115,116] including atrophic scars and other wounds [117,118], bone defects [119,120], ligaments and tendons [121,122], menisci [123], and cartilage [124,125,126,127].

One issue that remains to be resolved regarding the use of EV in MSK tissue repair protocols is whether a single dose of EVs at the time of implantation of a population of EV is sufficient to enhance repair, or whether a continual source of EV via implanted MSC or iPSC are required to optimize repair. Once dense connective tissue repair has been initiated, it may be difficult to administer repeat doses of EV that can get to endogenous cells that are surrounded by a dense ECM.

Both MSC and iPSC appear to function in tissue repair via release of EV [128,129,130,131]. Comparative proteomic profiling of EV from MSC and iPSC has revealed differences [128] possibly due to differences in origins of the cells as differences in EV cargos were also evident in MSC derived from different tissues [128]. In addition, EV derived from iPSC improved outcomes in a mouse diabetic wound healing model [129], so the EV may serve multiple functions in wound healing including modulating the inflammatory environment.

In summary, several types of cellular interventions have been used in attempts to enhance repair of injured or damaged tissues. While the results are encouraging for some of them, the outcomes are variable likely in part due to the complexity of the tissues involved, host factors such as age and genetics, as well as variation in the preparation and application of the intervention materials. However, issues related to the injury site are rarely mentioned.

## 3. Biomaterials and Scaffolds

The addition of free cells, whether they be MSC, iPSC, chondrocytes or other cell types to an injury site or a site damaged by disease without a mechanism to retain the cells at the site would not lead to high chance for success. Therefore, many studies have investigated a large variety of artificial matrices comprising various cell-compatible molecules that may be biodegradable (reviewed in [132,133]). Some of these have structure via bioprinting as cell-biomaterial composites (reviewed in [134,135,136,137,138,139]) or are amorphous and merely support the cells localizing to a specific site. Some also can contain growth factors or immunomodulating molecules. Nearly all of these artificial scaffolds are not functional regarding biomechanics but serve a more intermediate function for the cells to exert their abilities to express ECM molecules. With the emergence of machine learning and artificial intelligence tools, such cell-biomaterial composites are becoming more sophisticated and contain a more complex structure and functions (discussed in [140,141,142]).

In contrast to a purely artificial matrix/scaffold which can be used to envelop the cells to be implanted, other investigators have used a more natural ECM produced by the cells themselves [143,144,145]. The matrix generated following in vitro culture of synovium-derived MSC did not represent the matrix of natural articular cartilage, following implantations of this tissue engineered construct (TEC) into cartilage defects without any exogenous scaffold, or exogenous biomaterial. The implanted TEC evolved into articular cartilage-like material with layers similar to natural cartilage [146]. This TEC did not require any fixation as it adhered to the damaged tissue without fixation, likely due to its fibronectin content. Many other cell or cell-biomaterial composites require fixation or use of adhesive hydrogels (discussed in [147]).

One of the limitations of artificial biomaterials is that they do not have a template for the cells to build on in response to the biomechanical cues once implanted, but as discussed above such composites are becoming more complex in this regard. However, in the semi-natural biomaterial of the TEC system [144,145], the cells replace the implanted matrix with one that more closely resembles natural articular cartilage with a surface layer expressing PRG4, and a layered structure [146]. This may result from both the biology of the system as well as the biomechanical environment.

There are lessons to be learned from both cell-biomaterial composites and the somewhat natural ECM of the TEC approach. Both approaches have advantages and limitations, but they are leading to targeted and focused interventions for specific clinical indications. What is emerging is the likely need for personalized regenerative medicine approaches in response to some indications (discussed in [148]).

## 4. Successes and Challenges of Approaches Discussed

Many of the approaches discussed above have had varied success in preclinical and human studies. Often there is short term success, but the implanted cells or cell-biomaterial composites do not exhibit long-term success. In part this may have resulted from the preclinical models used for the studies, or there were unrealistic expectations built on incomplete knowledge. For the clinical studies, some of the limited success may be based on a lack of understanding of host variables that influence outcomes, along with applying cells and cell-biomaterial composites to conditions or diseases that limit the potential for success.

One example of success for long term outcomes in articular cartilage repair using stem/stromal cells is the TEC system discussed above (discussed in [144,145]). Based on a detailed analysis of large animal studies [149], and a focus on a limited fresh cartilage defect model, it was shown that the TEC approach led to good cartilage repair. This approach has now been successfully translated into human studies, again using cartilage defects as the focus. Implantation of autologous TEC into five patients has led to long-term (>5 years) cartilage repair [144]. Current multi-center studies are expanding the initial studies with larger cohorts.

While the above studies can likely be considered on the path to success, the focus has been on cartilage defects, and this is a defined subset of potential applications. However, as cartilage defects can progress to early OA and then more advanced OA, it remains to be observed whether the initial successes can be expanded into more diverse patient populations where the host environment is overtly altered. This consideration also likely applies to other applications, particularly in preclinical models where the cells and cell-biomaterial composites are applied very early after injury or in the disease process.

## 5. The “Other Half of the Equation”: Role of the Implantation Environments and Host Variables

When a connective tissue undergoes an injury from trauma, there is an acute reaction, leading to activation of the coagulation pathway, initiation of inflammation, and mobilization of a number of endogenous cell types and invasion of others, such as those of the blood. Thus, the environment after injury or development of a MSK disease would often lead to a local environment which is not optimal for initiating exogenous repair (Figure 2). Thus, optimizing in vivo functional outcomes may require not only the features of the implant materials but also the in vivo environment.

For example, after rupture of the intraarticular ACL, there can be the release of blood into the synovial fluid, degradation of the now immobilized ACL tissue and induction of an inflammatory response in the synovial space. The release of pro-inflammatory cytokines into the synovial fluid can lead to secondary impact on other tissues of the knee organ system. Exposure to cytokines such as IL-1 can decrease PRG4 expression by superficial zone cartilage cells, compromising the boundary lubrication of the cartilage [42]. Such inhibition could lead to loss of the articular cartilage surface and OA development [150]. Such cytokines could also impact the state of the synovium and Hoffa’s fat pad, a tissue that is highly innervated [151] that could become activated and contribute to loss of tissue integrity [152,153]. Surgery for implantation of autologous, allogenic or tissue-engineered ACL grafts could further result in inflammation and collateral damage to tissues.

OA is a chronic inflammatory joint disease (discussed in [154]) and various tissues in the joint can be altered, again including the synovium and Hoffa’s fat pad. This can result in several cytokines/adipokines being present in synovial fluid (SF). Therefore, the affected articular cartilage is no longer normal, nor are several other tissues of the organ system of a joint such as a knee. Implantation of a tissue-engineered material containing MSC, iPSC or chondrocytes would be subjected to exposure to pro-inflammatory mediators and risk subverting their function. For instance, one of the mediators present in SF is monocyte chemotactic protein-1 (MCP-1) [155]. Exposure of MSC to MCP-1 inhibits their differentiation to chondrogenesis [155]. Thus, the presence of MCP-1, or other cytokines, in synovial fluid could impact the functioning of MSC implanted in a cell-biomaterial composite. Therefore, controlling the local environment is likely critical for success in this regard.

Once injured, such as a defect generated in articular cartilage from sports or an accident, a connective tissue adapts to its new biological and biomechanical environment. As cartilage has poor healing capabilities, such adaptation does not lead to repair of the tissue. Therefore, the defect environment becomes nonfunctional and over time this disrupted integrity can lead to development of OA. Thus, implantation of a cell-biomaterial construct into the defect area after a period of time may not lead to effective integration with the adapted surface of the endogenous tissue. This could lead to sluffing of the implant, loss of implant integrity, or cell death and ultimate failure of the implant. Revitalizing the surfaces of the defect to remove the adapted tissue may be required to allow for better integration with the implanted constructs. This may also involve the transition of the endogenous response from an acute response to one that is considered chronic, a transition that alters the local environment to be less accommodating to an implant [156].

In summary, a number of interventions to improve the implant environment are likely required to enhance the opportunities for success. Most published studies in preclinical models usually implant a cell-biomaterial construct immediately after generation of a defect [143,149,157], a situation that does not reflect real life scenarios. Studies in domestic pigs have yielded very good results, but the implants still have some surface abnormalities [157]. However, some studies in humans with cartilage defects are treated with tissue engineered constructs withing a few weeks or months of the injury, and with good results [144,145]. However, studies with longer timeframes after the injury to articular cartilage after adaptation has occurred or OA developed, have not had high long-term success rates. Therefore, preclinical studies with larger animals such as the pig should be entertained where implants are put into defects at increasing times post-defect generation to determine whether there is an optimal time fame beyond which modifying the endogenous tissue and inflammatory environment is required to enhance success.

### 5.1. Learning from Skin Wound Healing

While skin is a connective tissue, it is in a unique environment being directly exposed to air, oxygen, and sunlight. During early fetal development, skin wounds heal with regeneration (reviewed in [158,159]) until a certain time point that appears to coincide with the onset of an inflammatory response, after which it heals with scar tissue. Skin injuries in young humans often heal with minimal scarring, while injuries incurred later in life demonstrate more scarring, indicating that healing is not static across the lifespan. It is quite challenging to mimic fetal wound healing to enhance adult skin wound healing [160]. However, healing of adult injuries to oral mucosa still leads to excellent repair/regeneration [161], due to reduced inflammation and regulation of myofibroblasts [161]. In a preclinical porcine model, it was shown that skin versus oral mucosa injuries heal very differently on the same animal [162]. Such findings indicate support for the concept that the injury environment is central to regulation of outcome and perhaps one will have to re-engage the fetal environment and conditions to better inform adult healing [163].

Skin wound healing can also be influenced by co-morbidities such as diabetes [164], nutrition [165], and other variables [156]. For skin wound healing that is impaired due to known and unknown factors, injury sites are often debrided to remove aberrant responses in the wound bed to initiate fresh healing responses [166,167,168,169]. This has also been shown for skin grafting after burn or other loss of large areas of skin [170,171]. Thus, preparing a fresh wound bed is essential for initiation of a functional healing response [156] or for the success of implantation of a graft. In some respects, this mimics what has been discussed above for repair of other tissues of the MSK system.

While the environment in the injured skin and the resulting healing process may be unique and different from environments such as within the injured knee, or perhaps other MSK injury sites, the principle of a well-prepared injury “bed” or site is needed to facilitate a good healing outcome can be extended to these other body locations.

### 5.2. Tendon Healing Lessons

While the above discussion has focused on general parameters that could impact the success of implanted materials to enhance repair success, there may also be variation in endogenous variables that also contribute to repair success. One situation that exemplifies this point has been reported regarding endogenous repair of ruptured Achilles tendons. Reports by Chen et al. [77,172,173] have identified biomarkers that are apparent 4–7 days post-injury that predict good versus poor outcome at 1-year post-injury. Thus, through genetic or stochastic factors, some individuals may generate more effective repair than others. If genetic variables influence outcomes, these findings offer the opportunity to better understand how to improve outcomes. Furthermore, if such findings can be shown to extend to other wound healing situations, then the extent of success following implantation of cells-biomaterial composites may also be influenced by such currently unknown host variables.

Such considerations may lead to study designs that are more individualized rather than those that focus on group comparisons. Current group comparisons in defining the efficacy of interventions would be strongly influenced by the heterogeneity discussed above as the intervention may not improve the outcomes for the naturally good outcome subpopulations and mainly impact the healing of the subset with naturally poor outcomes. Defining success would be impacted by the contributions of these subsets in any particular clinical trial. Thus, pre-screening would be required before entry into a trial, or an N of 1 trial design may be required (discussed in [174]).

## 6. Timing of the Intervention with a Cell-Biomaterial Composite

Current medical paradigms often wait to perform somewhat experimental procedures once all of the established approved procedures have been tried and not yielded success. In a situation such as in knee OA, this means that patients are diagnosed, prescribed over the counter pain drugs, and perhaps exercise protocols but then often left to their own devices until the pain and/or disability becomes too severe. They are then often provided with braces and stronger medications. Ultimately, when the condition becomes very severe and by MRI there is very little cartilage remaining, they undergo joint replacement. Some may be offered microfracture procedures [175,176,177] before joint replacement or biologic intervention with stem cells or chondrocytes. Such patients most often have considerable cartilage loss, bone changes, and other joints may become involved due to the development of altered gait. Adaptations to an injury or disease within a joint can lead to time-dependent changes that would likely impact any cell therapy intervention [11]. If one assumes that the implanted MSC or iPSC are going to replace the cartilage, then this approach may work if they are implanted into a suitable environment as discussed above. However, if the implanted stem or iPSC cells serve as facilitators of endogenous cell enhancement of repair then this approach may not lead to the success that is hoped for. In the studies by Nakagawa et al. [108], the implanted MSC and iPSC had disappeared from the repair tissue and been replaced by endogenous cells. Thus, if endogenous cells are recruited and enhanced in their ability to repair damaged tissues, then one would want to initiate repair when there were sufficient numbers of endogenous cells available to optimize repair. This approach may also alleviate development of aspects of adverse environmental conditions that would need to be counteracted. Thus, inverting the standard pyramid of practice would potentially offer enhanced opportunity for success of MSC and iPSC approaches to enhance MSK tissue repair.

## 7. Modifying the Host Environment to Enhance Success of Repair Following Implantation of Cell-Biomaterial Constructs

A number of variables or factors contribute to the outcome after an injury to a MSK tissue (see Graphical Abstract). Some of these are modifiable while others are more difficult to influence and others likely cannot be influenced at all.

Some host variables such as comorbidities may be challenging to modify but other optional variables such as smoking cessation, restricting alcohol use, and nutrition can be altered. In diabetics, good glucose control may diminish aspects of adversity to good wound healing, and in cardiovascular diseases, adhering to medication schedules may also diminish adverse influences.

Regarding the adverse injury environment for a tissue of the MSK system, likely one of the initial targets for intervention is the inflammatory response. Development of a chronic inflammatory state at an injury site may be localized to the injured tissue (i.e., a tendon) but if the injured tissue is a component of a joint, then tissues other than the one injured could become involved also. Thus, if cartilage of the knee is damaged, then the inflammation could also involve the synovial membrane, Hoffa’s fat pad, or the joint capsule. While the previous discussion indicated that the use of corticosteroids immediately after injury was beneficial, such drugs cannot be used chronically and may damage tissues. An alternative to the use of such drugs could be the use of EV with a cargo optimized for anti-inflammatory impact. Using a standardized EV protocol, such EV could be used repeatedly and injection of such products into the synovial space after a joint injury could impact a variety of tissues. PRP could also be used as ~10% of plasma proteins are proteinase inhibitors and platelets contain anabolic components.

A second target should be to debride the injury site, particularly if there has been a time lapse since the time of injury and the point of intervention (discussed in [178]). For knee tissues such as those with cartilage defects, the area around the edge of the defect site should be abraided to remove any tissue damaged by the mechanical loading of the cartilage. If the defect is now down to the bone, perhaps some of the altered bone should be removed and the repair would involve an osteochondral defect. As discussed earlier in this article, for chronic skin wounds, the debridement to remove damaged tissue, minimizing infection, and enhancing neovascularization is critical to remove impediments to healing.

Further to those two targets, additional alteration of a wound/injury environment may depend on location of the injury [179], whether the target site is very large when one may have to consider transplantation of tissue as well as a cell or cell-scaffold intervention. Additional considerations may also be the biomechanical environment since loss of mechanical stimulation may also lead to atrophy of residual tissue, as well as whether the patient has neuropathy that may compromise the healing of some tissues.

As it is likely that many changes in the local environment that contribute to the development of impediments to good repair are the result of chronicity or length of time since the injury to the time of implantation of cells or cell-scaffold constructs, to minimize the need to interfere with their impact on healing, one should consider performing the intervention as early as possible and not consider it to be the “intervention of last resort”.

Unfortunately, the above consideration cannot be met for many patients and thus preliminary studies with a relevant preclinical model is required. Such models allow for assessment of variables associated with different stages of the post-injury and post-disease environment. As outlined in Table 1, the pig is likely the model of choice for such studies. Pig models have been used to study repair of porcine cartilage repair [143,146,180,181], meniscus repair [182,183], bone repair [184], intervertebral disc repair [185,186], ligament repair [187], and skin repair [188,189] so porcine models are used for the study of repair of multiple MSK tissues. While studies with pigs may be expensive, the constellation of features, including the good potential for translation to humans, overcomes this limitation (discussed in [190]). In contrast, and based on a review of literature, studies with rodents appear to have a much lower translational potential.

Central to all of the above initiatives will be the development of optimized biomechanical loading protocols for specific applications. As tissues of the MSK system all function in mechanically active environments, tissue-specific loading protocols as repair tissue is deposited and matures are likely critical for achieving optimal functionality. For example, increasing loading as an excisional injury to the MCL leads to directional organization of the collagen fibers [214,215]. Without an appropriate loading environment, or no loading, it is likely that any deposited ECM will mature and become more fibrotic with amorphous collagen and other matrix molecules lacking functional organization and composition.

Finally, the rate of progress in the development of interventions with high potential for translation to human patients could benefit from changes to how such research is performed, from primarily a single laboratory-based approach to a more consortium-based approach involving standardized protocols, shared reagents, and a collaborative approach. This type of research approach could involve researchers, clinicians, veterinarians, and engineers and is an approach that could enhance the realization of the potential of MSC, iPSC, EV, and others much more rapidly than the previous >35 years.

## 8. Conclusions

**Figure 3:**
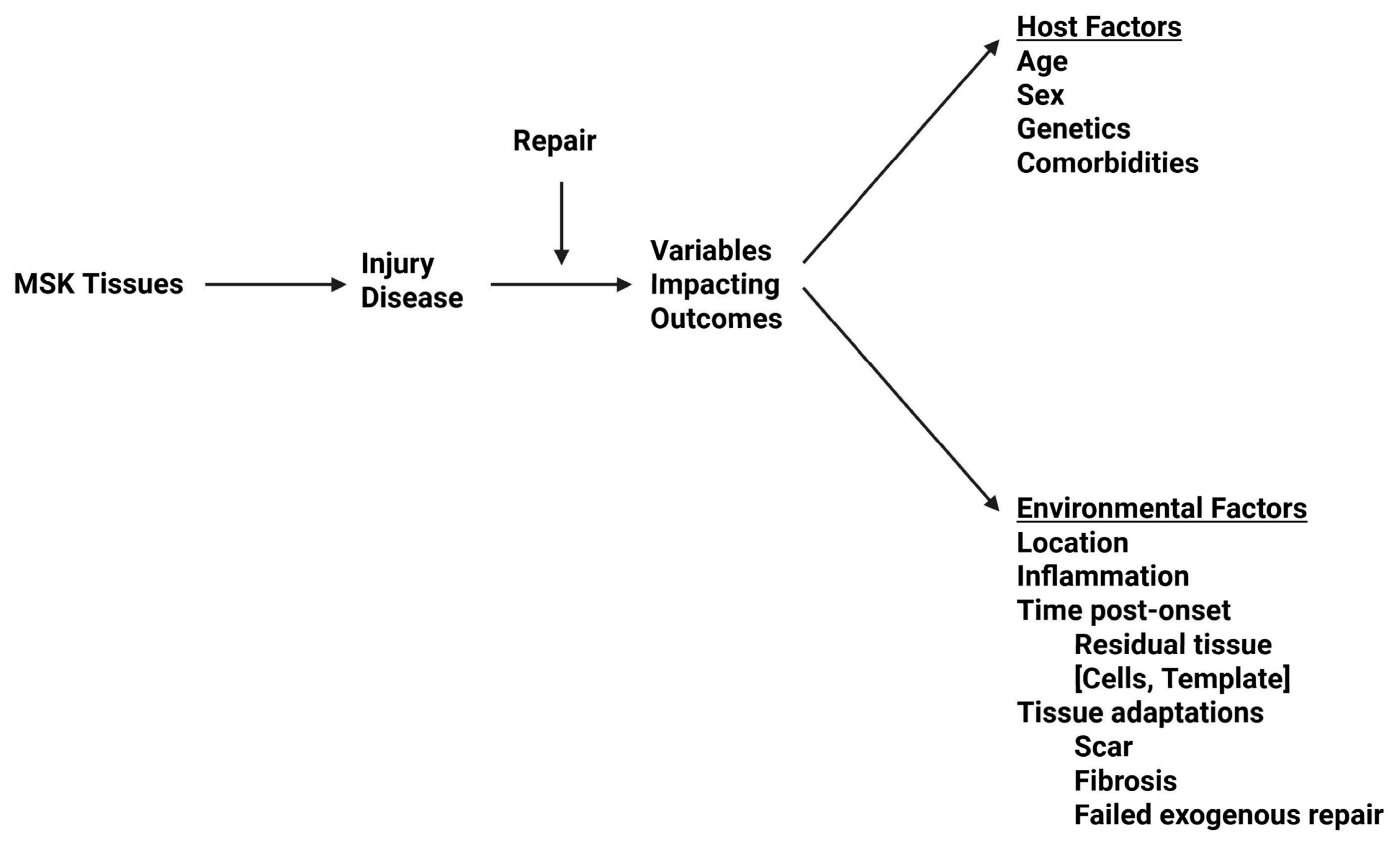
Outline of host and environmental factors that can potentially impact outcomes following implantation of MSC or iPSC composites. The complexity of these factors may require individualized clinical designs to test the effectiveness of cell therapy interventions.

Therapies to enhance healing and, potentially, the regeneration of tissues compromised by injury and/or disease requires an optimized protocol for specific applications. When such protocols involve cellular therapies, a number of variables go in the choices made, along with the carrier that maintains the cells or cell products at the appropriate site. However, the successful application of an optimized protocol also requires optimizing the implantation site to allow for the intervention to attain its potential. Not only the implantation site must be considered, but also the state of the host and variables related to the individual (Figure 3).

Much research effort over the past decades has explored the potential of both mesenchymal stem/stromal cells (now as suggested by Caplan [98], Medicinal Signaling Cells), iPSC and potent products of such cells (i.e., EV) to improve healing. While progress is being made in their use, along with smart biomaterials, there have been many challenges and the rate of progress for many applications has not lived up to expectations. As discussed, some successful translational efforts continue to support the potential of such approaches.

However, it can also be concluded that efforts to optimize the environments that cell therapies are implanted have not kept pace with the advances in cell therapies, and this has likely contributed to the lack of progress for long-term repair/regeneration of some very clinically relevant tissues. Research on optimizing the environments for implantation of cell–matrix composites should be a focus going forward, with the ultimate integration of the two arms of investigation.

Given the complexity of the natural structure and composition of some of these tissues to be targeted, unique features of their locations, and host variables (Figure 3), some of the future directions may require a personalized approach to effective long-term repair. Whether regeneration of damaged tissues with very complex biological and biomechanical environments can be achieved remains to be seen.

## Figures and Tables

**Table 1:** Advantageous Characteristics and Uses of Porcine Preclinical Models for MSK Tissue Repair.

Characteristic/Feature/Use	Reference
Genome sequence known	[191]
	[192]
Can be genetically manipulated	[193,194,195,196]
Several genetically distinct strains available	[197,198]
Physiology similar to humans	[192]
Some porcine tissues transplanted to humans	[199]
Tissues can be readily assessed biomechanically Skin (normal and scar)	[200,201]
Ligament (normal and scar)	[202,203]
Cartilage	[204]
Porcine skin similar to human skin	[205,206]
Can be nutritionally manipulated by diet	[207]
Used in wound healing and tissue repair research	[91,143,208,209]
Used for proteomic studies	[210]
Relevant co-morbidities can be induced (i.e., diabetes)	[180,211,212,213]
Documented tissue repair translation potential to humans	[144,145]

## References

[1] Albersheim M., Fedje-Johnston W., Carlson C., Arnoczky S.P., Toth F., Shea K., Harper L., Rendahl A., Tompkins M. (2023). Cell count and cell density decrease as age increases in cadaveric pediatric medial menisci. Arthrosc. Sports Med. Rehabil..

[2] Frank C.B. (2004). Ligament structure, physiology and function. J. Musculoskelet. Neuronal Interact..

[3] Khan I.M., Redman S.N., Williams R., Dowthwaite G.P., Oldfield S.F., Archer C.W. (2007). The development of synovial joints. Curr. Top. Dev. Biol..

[4] Bursac P., York A., Kuznia P., Brown L.M., Arnoczky S.P. (2009). Influence of donor age on the biomechanical and biochemical properties of human meniscal allografts. Am. J. Sports Med..

[5] Rattner J.B., Matyas J.R., Barclay L., Holowaychuk S., Sciore P., Lo I.K.Y., Shrive N.G., Frank C.B., Achari Y., Hart D.A. (2011). New understanding of the complex structure of the knee menisci: Implications for injury risl ad repair potential for athletes. Scand. J. Med. Sci. Sports.

[6] Lo I.K., Chi S., Ivie T., Frank C.B., Rattner J.B. (2002). The cellular matrix: A feature of tensile bearing dense soft connective tissues. Histol. Histopathol..

[7] Lo I.K.Y., Ou Y., Rattner J.-P., Hart D.A., Marchuk L.L., Frank C.B., Rattner J.B. (2002). The cellular networks of normal ovine medial collateral and anterior cruciate ligaments are not accurately recapitulated in scar tissue. J. Anat..

[8] Bruehlman S.B., Rattner J.B., Matyas J.R., Duncan N.A. (2002). Regional variations in the cellular matrix of the annulus fibrosus of the intervertebral disc. J. Anat..

[9] Hellio Le Graverand M.P., Sciore P., Eggerer J., Rattner J.P., Vignon E., Barclay L., Hart D.A., Rattner J.B. (2001). Formation and phenotype of cell clusters in osteoarthritic meniscus. Arthritis Rheum..

[10] Radin E.L., Burr D.B., Caterson B., Fyhrie D., Brown T.D., Boyd R.D. (1991). Mechanical determinants of osteoarthrosis. Semin. Arthritis Rheum..

[11] Frank C.B., Shrive N.G., Boorman R.S., Lo I.K.Y., Hart D.A. (2004). New perspectives on bioengineering of joint tissues: Joint adaptation creates a moving target for engineering replacement tissues. Ann. Biomed. Eng..

[12] Loeser R.F., Goldring S.R., Scanzello C.R., Goldring M.B. (2012). Osteoarthritis: A disease of the joint as an organ. Arthritis Rheum..

[13] Di Martino A., Barile F., D’Agostino C., Castafaro V., Cerasoli T., Mora P., Ruffilli A., Traina F., Faldini C. (2024). Are there gender-specific differences in hip and knee cartilage composition and degeneration? A systematic literature review. Eur. J. Orthop. Surg. Traumatol..

[14] Gilmer G., Crasta N., Tanaka M.J. (2025). The effect of sex hormones on joint ligament properties: A systematic review and meta-analysis. Am. J. Sports Med..

[15] Capuana E., De Luca A., Costa V., Raimondi L., Bellavia D., Brucato V., Giavaresi G., La Crrubba V. (2026). Advanced biomaterial-based in vitro osteoarthritis models: Integrating sex as a biological variable in hormonal, subchondral bone, and mechanobiological pathways. J. Funct. Biomater..

[16] Park S.K., Stefanyshan D.J., Loitz-Ramage B., Hart D.A., Ronsky J.L. (2009). Changing hormone levels during the menstrual cycle affect knee laxity and stiffness in healthy female subjects. Am. J. Sports Med..

[17] Hart D.A. (2023). Sex differences in musculoskeletal injury and disease risks across the lifespan: Are there unique subsets of females at higher risk than males for these conditions at distinct stages of the life cycle?. Front. Physiol..

[18] Manfre M.G., Richman E.H., Araujo-Espinoza G., Frank R.M. (2025). Anterior cruciate ligament injuries in female athletes. Clin. Sports Med..

[19] Thompson K., Quinn C. (2026). Epidemiology of sports-related injuries in female athletes. Clin. Sports Med..

[20] Dumas G.A., Reid J.G. (1997). Laxity of knee cruciate ligaments during pregnancy. J. Orthop. Sports Phys. Ther..

[21] Weckerly M.L., Feingold C.L., Singh N.K., Fatehi M.K., Abrahams R., Mirzai D.A., Cohen A., Cooper A. (2026). Pregnancy and lactation associated osteoporosis of the hip: A systematic review. Calcif. Tissue Int..

[22] Issa Z., Saad R., Lama Y., Ammar S., Akki V., Kovacs C.S., Fuleihan G.E.-H., Chakhtoura M. (2026). Pregnancy and lactation associated osteoporosis: A systematic review. Calcif. Tissue Int..

[23] Tuzun S., Aygun E. (2026). Pregnancy and lactations-associated osteoporosis: Insights from an onlie self-reported patient survey. Arch. Osteoporos..

[24] Kalkwarf H.J., Specker B.L. (1995). Bone mineral loss during lactation and recovery after weaning. Obstet. Gynecol..

[25] Pearson D., Kaur M., San P., Lawson N., Baker P., Hosking D. (2004). Recovery of pregnancy mediated bone loss during lactation. Bone.

[26] Bliddal M., Pottegard A., Kirkegaard H., Olsen J., Jorgensen J.S., Sorensen T.I.A., Dreyer L., Nohr E.A. (2016). Association of pre-pregnancy body mass index, pregnancy-related weight changes, and parity with the risk of developing degenerative musculoskeletal conditions. Arthritis Rheumatol..

[27] Gungor E., Gungor Z.K. (2024). Obstetric-related lower back pain: The effect of number of pregnancy on development of chronic lower back pain, worsening of lumbar disc degeneration and alteration of lumbar sagittal balance. J. Orthop. Surg. Res..

[28] Hart D.A. (2022). Sex differences in biological systems and the conundrum of menopause: Potential commonalities in post-menopausal disease mechanisms. Int. J. Mol. Sci..

[29] Wang S., Wang H., Liu W., Wei B. (2019). Identification of key genes and pathways associated with sex differences in osteoarthritis based on bioinformatics analysis. Biomed. Res. Int..

[30] Hart D.A., Nakamura N., Shrive N.G. (2021). Perspective: Challenges presented for regeneration of heterogeneous musculoskeletal tissues that normally develop in unique biomechanical environments. Front. Bioeng. Biotechnol..

[31] Vogel K.G. (2003). Tendon structure and response to changing mechanical load. J. Musculoskelet. Neuronal Interact..

[32] Vogel K.G., Koob T.J. (1989). Structural specialization in tendons under compression. Int. Rev. Cytol..

[33] Blevins F.T., Djurasovic M., Flatow E.L., Vogel K.G. (1997). Biology of the rotator cuff tendon. Orthop. Clin. N. Am..

[34] Fallon J., Blevins F.T., Vogel K., Trotter J. (2002). Functional morphology of the supraspinatus tendon. J. Orthop. Res..

[35] Assimakopoulos A.P., Katonis P.G., Agapitos M.V., Exachou E.I. (1992). The innervation of the human meniscus. Clin. Orthop. Relat. Res..

[36] Gray J.C. (1999). Neural and vascular anatomy of the menisci of the human knee. J. Orthop. Sports Phys. Ther..

[37] Ashinsky B.G., Guillbrand S.E., Wang C., Bonnevie E.D., Han L., Mauck R.L., Smith H.E. (2021). Degeneration alters structure-function relationships at multiple length-scales and across interfaces in human intervertebral discs. J. Anat..

[38] Armiento A.R., Stoddart M.J., Eglin D. (2018). Biomaterials for articular cartilage tissue engineering: Learning from biology. Acta Biomater..

[39] Xu W., Zhu J., Hu J., Xiao L. (2022). Engineering the biomechanical microenvironment of chondrocytes towards articular cartilage tissue engineering. Life Sci..

[40] Wu G., Chen S., Li Q., Zhang M., Cao F., Wei J., Guo L., Li P., Wei X., Zhang Q. (2025). Mechanical cues enhance chondrocyte function: Insights from mechanoreception, regulation, and biological responses. Mechanobiol. Med..

[41] Xie P., Sun A.R., Gao F., Li L., Crawford R., Prasadam I. (2026). Resolving microenvironment complexity and cellular heterogeneity in osteoarthritis via spatial transcriptomics. Osteoarthr. Cartil..

[42] Jones A.R.C., Flannery C.R. (2007). Bioregulation of lubricin expression by growth factors and cytokines. Eur. Cell Mater..

[43] Ignatyeva N., Gavrilov N., Timashev P.S., Medvedeva E.V. (2024). prg4-expressing chondroprogenitor cells in the superficial zone of articular cartilage. Int. J. Mol. Sci..

[44] Tian P., Suo Y., Guo L., Li P., Chen J., Zhang J., Ma Y., Zhang X., Wang C., Wei X. (2026). Moderate exercise mobilizes Prg4+ cells in the superficial zone of articular cartilage and maintains cartilage homeostasis. Biomed. J..

[45] Poole A.R., Kojima T., Yasuda T., Mwale F., Kobayashi M., Laverty S. (2001). Composition and structure of articular cartilage: A template for tissue repair. Clin. Orthop. Relat. Res..

[46] Hart D.A. (2026). Integrating mechanical loading, mechanotransduction, and biological responses in musculoskeletal tissues across the lifespan: Regulation influenced by cells, extracellular matrix and sex. Results Probl. Cell Differ..

[47] Hart D.A. (2025). Regulation of joint tissues and joint function: Is there potential for lessons to be learned regarding regulatory control from joint hypermobility syndromes?. Int. J. Mol. Sci..

[48] Syx D., Malfait F. (2024). Pathogenic mechanisms in genetically defined Ehlers-Danlos syndromes. Trends Mol. Med..

[49] Junge T., Larsen L.R., Juul-Kristensen B., Wedderkopp N. (2015). The extent and risk of knee injuries in children aged 9-14 with generalized joint hypermobility and knee joint hypermobility-the CHAMPS-study Denmark. BMC Musculoskeletal. Discord..

[50] Schmidt S., Leite C.B.G., Bumberger A., Franco D., Jacobs C.A., Richardson L., Paschos N., Goertz S., Berkson E., Asnis P. (2025). Survival of anterior cruciate ligament reconstruction in patients with Ehlers-Danlos syndrome: A comparison with anatomic risk factors in existing literature. Int. Orthop..

[51] Schmidt S., Leite C.B.G., Bumberger A., Franco D., Jacobs C.A., Lattermann C. (2025). Risk factors of patellofemoral instability in patients with hypermobilie Ehlers-Danlos syndrome. Arch. Orthop. Trauma Surg..

[52] Islam M., Chang C., Gershwin M.E. (2020). Ehlers-Danlos syndrome: Immunologic contrasts and connective tissue comparisons. J. Transl. Autoimmun..

[53] Dai W., Li Z., Zheng R., Geng X., Wu K., Chang T., Chu X. (2026). Targeting the pathological microenvironment in diabetic foot ulcers: Advances in drug delivery systems for improved healing. J. Biomed. Mater. Res. B Appl. Biomater..

[54] Ibrahim R.M., Houreld N.N. (2026). The impact of stem cell secretome, exosomes, and photobiomodulation on enhancing diabetic wound recovery. Biomed. Pharmacother..

[55] Stolarczyk A., Sarzynska S., Gondek A., Cudnoch-Jedrzejewska A. (2018). Influence of diabetes on tissue healing in orthopaedic injuries. Clin. Exp. Pharmacol. Physiol..

[56] Oyama Y., Ueki Y., Otagin K., Itagaki T., Iguchi J., Okawa K., Miyagi T., Sakai T., Kitabayashi H., Etisawa S. (2026). Association between Achilles tendon thickness as assessed by ultrasound and coronary artery disease severity in patients undergoing percutaneous coronary intervention. Angiology.

[57] Shu Y., Zhang Q., He X., Liu Y., Wu Y., Chen L. (2022). Fluoroquinolone-associated suspected tendonitis and tendon rupture: A pharmacovivgilance analysis from 2016–2021 based on the FAERS database. Front. Pharmacol..

[58] Jaschke M., Kolodziej L., Huebotter C., Hennecke T., Koppe D., Wilk A. (2026). An overview in tendon’s physiology, pathomorphology, and treatment options. Cell Tissue Res..

[59] Hartog P., Hinkle L.J., Mitchell U.H., Johnson A.W. (2026). Longitudinal ultrasound assessment of Achilles and patellar tendon morphology in national collegiate athletic association division 1 female gymnasts. J. Funct. Morphol. Kinesiol..

[60] Li J., Cao B., Yu S., Li C., Xue M., Xie Q., Luo C., Duan C., Li Z., Jin C. (2026). Diabetes-related metabolic osteoarthritis: Advanced glycation-collagen axis, cartilage stiffening, and biomaterials-based therapeutic strategies. Int. J. Nanomed..

[61] Zhong J., Yang G. (2026). Metabolic syndrome-osteoarthritis axis: Beyond mechanical loading toward a cardiometabolic OA phenotype. Biomed. Pharmacother..

[62] Al Mamun A., Shao C., Geng P., Wang S., Xiao J. (2025). Recent advances in the role of neuroregulation in skin wound healing. Burns Trauma.

[63] Zhang Y., Liu X., Wu G. (2026). Hydrogel-based neural engineering for skin wound healing. Front. Cell Dev. Biol..

[64] Ackermann P.W., Franklin S.L., Dean B.J.F., Carr A.J., Salo P.T., Hart D.A. (2014). Neuronal pathways in tendon healing and tendinopathy—Update. Front. Biosci..

[65] Niu Y., Li Q., Ding Y., Dong L., Wang C. (2019). Engineered delivery strategies for enhanced control of growth factor activities in wound healing. Adv. Drug Deliv. Rev..

[66] Nakamura N., Hart D.A., Boorman R.S., Kaneda Y., Shrive N.G., Marchuk L.L., Shino K., Ochi T., Frank C.B. (2000). Decorin antisense gene therapy improves functional healing of early rabbit ligament scar with enhanced collagen fibrillogenesis in vivo. J. Orthop. Res..

[67] Oneto P., Etulain J. (2021). PRP in wound healing applications. Platelets.

[68] Zhang C., Zhou H., Zhang Z., Liu P., Xue X., Zhang Z., Wang L., Jiang Y., Zhang C., Zhou H. (2025). The role of platelet-rich plasma in biomedicine: A comprehensive overview. iScience.

[69] Zhang Z., Zhao S., Hei P. (2026). Global trends in platelet-rich plasma research: A bibliometric review with hotspot visualization. Medicine.

[70] Burnham R., Smith A., Hart D. (2021). The safety and effectiveness of bone marrow concentrate injection for knee and hip osteoarthritis: A Canadian cohort. Regen. Med..

[71] Heard B.J., Barton K.I., Chung M., Achari Y., Shrive N.G., Frank C.B., Hart D.A. (2015). Single intra-articular dexamethasone injections immediately post-surgery in a rabbit model mitigates early inflammatory responses and post-traumatic osteoarthritis-like alterations. J. Orthop. Res..

[72] Sieker J.T., Ayturk U.M., Proffen B.L., Weissenberger M.H., Kiapour A.M., Murray M.M. (2016). Immediate administration of intraarticular triamcinolone acetonide after joint injury modulates molecular outcomes associated with early synovitis. Arthritis Rheumatol..

[73] Grodzinsky A.J., Wang Y., Kakar S., Vrahas M.S., Evans C.H. (2017). Intra-articular dexamethasone to inhibit the development of post-traumatic osteoarthritis. J. Orthop. Res..

[74] Kydd A.S., Reno C., Sorbetti J.M., Hart D.A. (2005). Influence of a single systemic corticosteroid injection on mRNA levels for a subset of genes in connective tissues of the rabbit knee: A comparison of steroid types and effect of skeletal maturity. J. Rheumatol..

[75] Kydd A.S., Achari Y., Lu T., Sciore P., Rattner J.B., Hart D.A. (2005). The healing rabbit medial collateral ligament of the knee responds to systemically administered glucocorticoids differently than the uninjured tissues of the same joint or the uninjured MCL: A paradoxical shift in impact on specific mRNA levels and MMP-13 protein expression in injured tissues. Biochim. Biophys. Acta.

[76] Yasui Y., Hart D.A., Sugita N., Chijimatsu R., Koisumi K., Ando W., Moriguchi Y., Shimomura K., Myoui A., Yoshikawa H. (2018). Time-dependent recovery of human synovial membrane mesenchymal stem cell function after high/-dose steroid therapy: Case report and laboratory study. Am. J. Sports Med..

[77] Chen J., Fu X., Ahmed A.S., Hart D.A., Zhou Z., Ackermann P.W. (2026). Systematic review of relevant biomarkers for human connective tissue repair and healing outcome: Implications for understanding healing processes and design of healing interventions. Adv. Wound Care.

[78] Fagbongbe O., Salam R., Amaral J.Z., Kushare I., McKay S.D., Kan J.H. (2026). MRI of pediatric meniscal retears following arthroscopic surgery: Diagnostic accuracy and common findings. Skelet. Radiol..

[79] Dagher D., Shah D., Kruse C., Khalik H.A., Saleh U., Hachem A.-I., Tokish J.M., Bedi A., Khan M. (2026). Structural augmentation in rotator cuff repair decreases the risk of retear: A systematic review and meta-analysis. Am. J. Sports Med..

[80] Brittberg M. (2025). Cartilage repair with autologous chondrocytes (ACI generations 1–4). Clin. Sports Med..

[81] Wen J., Syed B., Ansari U., Thomas V., Shehabat M., Akhtar M., Razick D., Kreulen C.D. (2025). Favorable short-term outcomes of matrix-associated autologous chondrocyte implantation for osteochondral lesions of the talus: A systematic review. Arthroscopy.

[82] Sciarretta F.V. (2025). Cell transplantation techniques for cartilage restoration. Sports Med. Arthrosc. Rev..

[83] Dhinsa B.S., Adesida A. (2012). Current clinical therapies for cartilage repair, their limitation, and the role of stem cells. Curr. Stem Cell Res..

[84] Caplan A.I. (2015). Adult mesenchymal stem cells: When, where and how. Stem Cells Int..

[85] Pittenger M.F., Discher D.E., Peault B.M., Phinney D.G., Hare J.M., Caplan A.I. (2019). Mesenchymal stem cell perspective: Cell biology to clinical progress. npj Regen. Med..

[86] Freeman B.T., Jung J.P., Olge B.M. (2015). Single-cell RNA-seq of bone marrow-derived mesenchymal stem cells reveals unique profiles of lineage priming. PLoS ONE.

[87] Zhang X., Doolittle M.L., Fan T., Tripathi U., Ng Y.E., Jiang X., Passos J.F., Jurk D., Monroe D.G., Tchkonia T. (2026). Senescent mesenchymal stromal cells differentially alter adipogenesis in adipose tissue, skeletal muscle, and bone marrow. Obesity.

[88] Kasim N.H.A., Govindasamy V., Gnanasegaran N., Musa S., Pradeep P.J., Srijaya T.C., Aziz Z.A.C.A. (2015). Unique molecular signatures influencing the biological function and fate of post-natal stem cells isolated from different sources. J. Tissue Eng. Regen. Med..

[89] Kittilukkana A., Nimkingratana P., Pruksakorn D., Takuathung M.N., Koonrungsesomboon N. (2026). Research landscape of stem cell applications in musculoskeletal tissue: A scoping review. Cells.

[90] Ando W., Kutcher J.J., Krawetz R., Sen A., Nakamura N., Frank C.B., Hart D.A. (2014). Clonal analysis of synovial fluid stem cells to characterize and identify stable mesenchymal stromal cell/mesenchymal progenitor cell phenotypes in a porcine model: A cell source with enhanced commitment to the chondrogenic lineage. Cytotherapy.

[91] Ando W., Heard B.J., Chung M., Nakamura N., Frank C.B., Hart D.A. (2012). Ovine synovial membrane-derived mesenchymal progenitor cells retain the phenotype of the original tissue that was exposed to in-vivo inflammation: Evidence for a suppressed chondrogenic differentiation potential of these cells. Inflamm. Res..

[92] Voos J.E., Moyal A., Furdock R., Caplan A.I., Bonfield T.L., Calcei J.G. (2025). Culture expansion alters human bone marrow-derived mesenchymal stem cell production of osteoarthritis-relevant cytokines and growth factors. Arthroscopy.

[93] Klyushnenkova E., Mosca J.D., Zernetkina V., Majumdar M.K., Beggs K.J., Simonetti D.W., Deans R.J., McIntosh K.R. (2005). T cell response to allogeneic human mesenchymal stem cells: Immunogenicity, tolerance, and suppression. J. Biomed. Sci..

[94] Patel J.C., Shukla M., Shukla M. (2025). From bench to bedside: Translating mesenchymal stem cell therapies through preclinical and clinical evidence. Front. Bioeng. Biotechnol..

[95] Hum C., Poliwonda J., Lalu M., Stewart D.J., Mei S.H.J., Walley K.R., Marshall J., Mendelson A.A., Fergusson D.A., English S. (2026). Safety of intravascular administration of umbilical-cord-derived mesenchymal stomal cells: An updated systematic review and meta-analysis. Stem Cells Transl. Med..

[96] Caplan A.I. (2017). New MSC: MSCs as pericytes are sentinels and gatekeepers. J. Orthop. Res..

[97] Hart D.A. (2022). One of the primary functions of tissue-resident pluripotent pericytes cells may be to regulate normal organ growth and maturation: Implications for attempts to repair tissues later in life. Int. J. Mol. Sci..

[98] Caplan A.I. (2017). Mesenchymal stem cells: Time to change the name?. Stem Cells Transl. Med..

[99] Takahashi K., Yamanaka S. (2006). Induction of pluripotent stem cells from mouse embryonic and adult fibroblast cultures by defined factors. Cell.

[100] Okita K., Ichisaka T., Yamanaka S. (2007). Generation of germline-competent induced pluripotent stem cells. Nature.

[101] Yamanaka S. (2026). Two decades of induced pluripotent stem cell research: From discovery to diverse applications. Cell Stem Cell.

[102] Nakagawa M., Koyanagi M., Tanabe K., Takahashi K., Ichisaka T., Aoi T., Okita K., Mochiduki Y., Takizawa N., Yamanaka S. (2008). Generation of induced pluripotent stem cells without Myc from mouse and human fibroblasts. Nat. Biotechnol..

[103] Ozsezer G., Tosunoglu B.D., Arslam Y.E. (2026). A review on nanotoxicology in stem cell research: Challenges and safety assessments. Methods in Molecular Biology.

[104] Nakamura A., Murata D., Fujimoto R., Tamaki S., Nagota S., Ikeya M., Toguchida J., Nakayama K. (2021). Bio-3D printing iPSC-derived human chondrocytes for articular cartilage regeneration. Biofabrication.

[105] Nakamura A., Zujur D., Nonaka T., Yoshizato H., Kashimoto S., Ikeya M., Nakayama K. (2026). Modulation of iPSC-derived NCMSC cell state by TD-198946 enhances scaffold-free cartilage biofabrication. Biofabrication.

[106] Husain H., Li M., Abrahante J.E., Mancipe N.C., Vegoe A., Chai Y.W., Lindborg B., Tompkins M., Ogle B., Larsen P.A. (2025). Consistent self-organized emergence of hyaline cartilage in human pluripotent stem cell-derived multi-tissue organoids. Stem Cells.

[107] Laagland L.T., Liyanage D.W.L.P., Desprat R., Riemers F.M., Warmerdam C.C., Soubeyrand M., Bensadoun P., Ito K., Milhavet O., Camus A. (2026). Disc-derived induced pluripotent stem cells and environmental cues for nucleus pulposus regeneration. Tissue Eng. Part A.

[108] Nakagawa S., Ando W., Shimomura K., Hart D.A., Hanai H., Jacob G., Chijimatsu R., Yarimitu S., Fijie H., Okada S. (2023). Repair of osteochondral defects: Efficacy of a tissue-engineered hybrid implant containing both human MSC and human iPSC-cartilaginous particles. npj Regen. Med..

[109] Zhao B., Chen Q., Zhang Q., Tian W., Chen T., Liu Z. (2024). Therapeutic potential of adipose-derived stem cell extracellular vesicles: From inflammation regulation to tissue repair. Stem Cells Res. Ther..

[110] Nagao Y., Kajiyama H., Yokoi A. (2026). A novel extracellular vesicle isolation method based on cellulose nanofiber sheets. Extracell. Vesicles Circ. Nucl. Acids.

[111] Khan M.F., Wajid H., Begum S. (2026). Mechanistic reprogramming by stem cell-derived extracellular vesicles: From cargo sorting to tissue regeneration. Tissue Cell.

[112] Dunstan I.K., Anthony D.C., Lugarini F., Idriss S. (2026). Therapeutic potential of stem cell-derived extracellular vesicles in aging and regeneration. Front. Aging.

[113] Yang H., Zhao T., Zhao P., Cao C., Fang F., Liu X. (2026). Mechano-regulated extracellular vesicles: Key mediators in disease pathogenesis and emerging therapeutic avenues. Biomaterials.

[114] Hanai H., Hart D.A., Jacob G., Shimomura K., Ando W., Yoshioka Y., Ochiya T., Nakagawa S., Nakamura M., Okada S. (2023). Small extracellular vesicles derived from human adipose-derived mesenchymal stromal cells cultured in a new chemically-defined contaminate-free media exhibit enhanced biological and therapeutic effects on human chondrocytes in vitro and in a mouse osteoarthritis model. J. Extracell. Vesicles.

[115] Li Z., Hou D., Tang Z., Xiong L., Yan Y. (2024). The potential role of stem cells-derived extracellular vesicles in the treatment of musculoskeletal system diseases. Cell Bio. Int..

[116] Izquierdo S.G., Pilar A.F., Rios J.L., Lim K.S., Toh W.S., Liu C., Gimona M., Gawlitta D., Man K. (2025). Advances in extracellular vesicle-based nanomedicine for regenerative orthopaedics. J. Nanobiotechnol..

[117] Sarcinella A., Femmino S., Brizzi M.F. (2023). Extracellular vesicles: Emergent and multiple sources in wound healing treatment. Int. J. Mol. Sci..

[118] Jafarzadeh A., Hoseini S.S., Behrangi E., Rochaninasab M., Goodarzi A. (2026). Regenerative medicine for atrophic scars: A systematic review of extracellular vesicles, conditioned media, stromal vascular fraction and mesenchymal stem cells. Aesthetic Plast. Surg..

[119] Emami A., Arabpour Z., Izadi E. (2025). Extracellular vesicles: Essential agents in critical bone defect repair and therapeutic enhancement. Mol. Biol. Rep..

[120] Staubli F., Zhou Y., Vader P., Hofmann S., Bergsma J.E., Gawlitta D., Man K. (2026). Harnessing extracellular vesicles for endochondral bone regeneration: Mechanisms and applications. Acta Biomater..

[121] Lu V., Tennyson M., Zhang J., Khan W. (2021). Mesenchymal stem cell-derived extracellular vesicles in tendon and ligament repair-a systematic review of in vivo studies. Cells.

[122] Kasula V., Padala V., Gupta N., Doyle D., Bagheri K., Antastasio A., Adams S.B. (2024). The use of extracellular vesicles in Achilles tendon repair: A systematic review. Biomedicines.

[123] Cheung C., Li Z., Jo S.-H., Lee S.-H., Ko J., Heo S.C. (2026). Dynamic mechanical loading reprograms meniscus cell-derived extracellular vesicles to enhance their regenerative potency. bioRxiv.

[124] Conforti C., Kimbrough D.W., Patel N., Ley M.B.R.G., Flores J.M., Correa D., Kaplan L.D., Best T.M., Kouroupis D. (2025). Characterizing the long non-coding RNA profile of endometrial mesenchymal stem/stromal cell-derived extracellular vesicles and their anti-inflammatory role in osteoarthritis. Int. J. Mol. Sci..

[125] Chang L.-H., Chuang S.-C., Wu S.-C., Fu Y.-C., Chen J.-W., Wu C.-W., Lin Y.-S., Liu C.-Y., Chung Y.-H., Chang J.-K. (2025). Comparison of miRNA profiles of exosomes derived from human iPSCs, ADSCs, and BMSCs and effects on chondrocyte function. Bone Jt. Res..

[126] Kugaudaite G., Bakutyte J., Bagdonas E., Vaiculeviciute R., Talaikis M., Miksiunas R., Lebedis I., Pachaleva J., Kvederas G., Krugly E. (2026). Menstrual blood-derived mesenchymal stromal cell extracellular vesicles stimulate chondrocytes and cartilage extracellular matrix synthesis in vitro. Sci. Rep..

[127] Maruiwa Y., Niki Y., Mabuchi Y., Takeuchi O., Kuronuma S., Fukuda Y., Fujie A., Oya A., Kobayashi S., Nakamura M. (2026). Extracellular vesicles derived from adipose-derived mesenchymal stem/stromal cells prevent synovial inflammation, and attenuate cartilage degeneration in rodent osteoarthritis. Regen. Ther..

[128] Gupta S., Krishnakumar V., Soni N., Rao E.P., Banerjee A., Mohanty S. (2022). Comprative proteomic profiling of small extracellular vesicles derived from iPSCs and tissue specific mesenchymal stem cells. Exp. Cell Res..

[129] Levy D., Abadchi S.N., Shababi N., Ravari M.R., Pirolli N.H., Bergeron C., Obiorah A., Mokhtari-Esbuie F., Gheshlaghi S., Abraham J.M. (2023). Induced pluripotent stem cell-derived extracellular vesicles promote wound repair in a diabetic mouse model via an anti-inflammatory immunomodulatory mechanism. Adv. Healthc. Mater..

[130] Kmiotek-Wasylewska K., Labedz-Maslowska A., Bobis-Wozowicz S., Karnas E., Noga S., Sikula-Stryjewska M., Woznicka O., Madeja Z., Dawn B., Zuba-Surma E.K. (2024). Induced pluripotent stem cell-derived extracellular vesicles enriched with miR-126 induce proangiogenic properties and promote repair of ischemic tissue. FASEB J..

[131] Kawasumi R., Kawamura T., Yamashita K., Tominaga Y., Harada A., Ito E., Takeda M., Kita S., Shimomura I., Miyagawa S. (2024). Systemic administration of induced pluripotent stem cell-derived mesenchymal stem cells improves cardiac function through extracellular vesicle-mediated tissue repair in a rat model of ischemic cardiomyopathy. Regen. Ther..

[132] Zhang B., Zhang M., Jiang C., Yan C., Pan Y., Meng F. (2026). Engineered polysaccharide scaffolds for cartilage regeneration: Mechanisms, functionization, and clinical prospects. Colloids Surf. B. Biointerfaces.

[133] Maeso L., Antezana P.E., Municoy S., Zarate J., Lertxundi U., Mishra Y.K., Dolashahi-Pirouz A., Desimone M.F., Orive G. (2026). Recent advances in the use of gelatin as a biomaterial for tissue engineering. Int. J. Pharm..

[134] Li Y.-C., Shao B., Ye K., Liu X., Wang H.-D., Sun C.-L., Xiao Y.-Y., Wang H. (2026). 3D bioprinting and mesenchymal stem cells: A bibliometric analysis of emerging trends and advancements. Cell Transplant..

[135] Dutta S.D., Ganguly K., Patil T.V., Randhawa A., Lim K.-T. (2023). Unraveling the potential of 3D bioprinted immunomodulatory materials for regulating macrophage polarization: State-of-the-art in bone and associated tissue regeneration. Bioact. Mater..

[136] Talouki P.Y., Tamimi R., Mahmoodi M. (2026). Engineering the cartilage niche: Mimicking the microenvironment for functional outcomes. Tissue Cell.

[137] Jigar V., Nensi R., Puja V., Prajapati B., Panraksa P., Singh S., Chittasupho C. (2026). Bioinspired polymeric scaffolds for improvement of angiogenesis and tissue engineering: A review. Polymer.

[138] Demri N., Descroix S., Wilhelm C. (2026). Engineering anisotropic tissues: From structured scaffolds to magnetic actuation. Mater. Today Bio.

[139] Khatibi A., Khazaei M., Zamani S., Jaf M.A., Rezakhani L. (2026). 3D bioprinting technologies and biomaterial-based scaffolds for wound healing: Insights into decellularized tissue-derived bioinks. Regen. Ther..

[140] Maeso L., Nadimifor M., Biswas S., Roy S., Gaharwar A.K., Dolatshahi-Pirouz A., Orive G. (2026). Theranostic platforms for skin tissue engineering: Bridging healing and real-time monitoring. Biomater. Adv..

[141] Zhang H., Feng J., Zhu J., Deng Y., Zou R., Fu L., Jiang H. (2026). The application of tissue engineering in cartilage regeneration: Technological advances and future challenges. Front. Bioeng. Biotechnol..

[142] Das I.J., Samal H.B., Nayak P., Swain S.S. (2026). Machine learning in tissue engineering: A comprehensive review of applications, challenges, and prospects. J. Biomater. Sci. Polym. Ed..

[143] Ando W., Tateishi K., Hart D.A., Katakai D., Tanaka Y., Nakata K., Hashimoto J., Fujie H., Shino K., Yoshikawa H. (2007). Cartilage repair using an in vitro generated scaffold-free tissue-engineered construct derived from porcine synovial mesenchymal stem cells. Biomaterials.

[144] Shimomura K., Ando W., Hart D.A., Yonetani Y., Horibe S., Nakamura N. (2023). Five-year outcomes after implantation of a scaffold-free tissue-engineered construct generated from autologous synovial mesenchymal stromal cells for repair of knee chondral lesions. Orthop. J. Sports Med..

[145] Shimomura K., Ando W., Hart D.A., Nakamura N. (2024). A novel scaffold-free mesenchymal stem cell-derived tissue engineered construct for articular cartilage restoration-from basic to clinic. Regen. Ther..

[146] Fujie H., Nansai R., Ando W., Shimomura K., Moriguchi Y., Hart D.A., Nakamura N. (2015). Zone-specific integrated cartilage repair using a scaffold-free tissue engineered construct derived from allogenic synovial mesenchymal stem cells: Biomechanical and histological assessments. J. Biomech..

[147] Jo H., Lee S.S. (2026). Adhesive hydrogels as fixation and regeneration platforms in cartilage surgery: Rethinking scaffold-tissue integration from a clinical perspective. Int. J. Mol. Sci..

[148] Arciola C.R., Panichi V., Bua G., Costantini S., Bottau G., Ravaioli S., Capponi E., Campoccia D. (2026). Smart healing for wound repair: Emerging multifunctional strategies in personalized regenerative medicine and their relevance to orthopedics. Antibiotics.

[149] Shimomura K., Ando W., Tateishi K., Nansai R., Fijie H., Hart D.A., Kohda H., Kita K., Kanamoto T., Mae T. (2010). The influence of skeletal maturity on allogenic synovial mesenchymal stem cell-based repair of cartilage in a large animal model. Biomaterials.

[150] Takahata Y., Hagino H., Kimura A., Urushizaki M., Yamamoto S., Wakamori K., Murakami T., Hata K., Nishamura R. (2022). Regulatory mechanisms of *prg4* and *gdf5* expression in articular cartilage and functions in osteoarthritis. Int. J. Mol. Sci..

[151] Draghi F., Ferrozzi G., Urciuoli L., Bortolotto C., Bianchi S. (2016). Hoffa’s fat pad abnormalities, knee pain, and magnetic resonance imaging in daily practice. Insights Imaging.

[152] Heard B.J., Solbak N.M., Chung M., Achari Y., Shrive N.G., Frank C.B., Hart D.A. (2016). The infrapatellar fat pad is affected by injury induced inflammation in the rabbit knee: Use of dexamethasone to mitigate damage. Inflamm. Res..

[153] Zhou S., Maleitzke T., Geissler S., Hildebrandt A., Fleckstein F.N., Neimann M., Fischer H., Perka C., Duda G.N., Winkler T. (2022). Source and hub of inflammation: The infrapatellar fat pad and its interactions with articular tissues during knee osteoarthritis. J. Orthop. Res..

[154] Hart D.A. (2022). Osteoarthritis as an umbrella term for different subsets of humans undergoing joint degeneration: The need to address the differences to develop effective conservative treatment and prevention strategies. Int. J. Mol. Sci..

[155] Harris Q., Seto J., O’Brien K., Lee P.S., Kondo C., Heard B.J., Hart D.A., Krawetz R.J. (2013). Monocyte chemotactic protein-1 inhibits chondrogenesis of synovial mesenchymal progenitor cells: An in vitro study. Stem Cells.

[156] Falanga V. (2004). The chronic wound: Impaired healing and solutions in the context of wound bed preparation. Blood Cells Mol. Dis..

[157] Ando W., Fujie H., Moriguchi Y., Nansai R., Shimomura K., Hart D.A., Yoshikawa H., Nakamura N. (2012). Detection of abnormalities in the superficial zone of cartilage repaired using a tissue-engineered construct derived from synovial stem cells. Eur. Cell Mater..

[158] Gomes M.L.N.P., Krijnen P.A., Middelkoop E., Niessen H.W.M., Boekema B.K.H.L. (2025). Fetal skin wound healing: Key extracellular matrix components and regulators in scarless healing. J. Investig. Dermatol..

[159] Weng T., Zhang L., Liu Y., Zhao X., Yue X. (2025). Advances in tissue engineering and biomaterials for minimizing wound scarring: Current status and future challenges. J. Funct. Biomater..

[160] Chakrabarti S., Ahmed R.K., Nath S., Nandi A., Pathak M.P. (2026). Scar-free wound healing: Advances in biomaterial-based formulations, smart dressings and bioactive therapeutics-a comprehensive review. Biochem. Biophys. Res. Commun..

[161] Mak K., Manji A., Gallant-Behm C., Wiebe C., Hart D.A., Larjava H., Hakkinen L. (2009). Scarless healing of oral mucosa is characterized by faster resolution of inflammation and control of myofibroblast action compared to skin wounds in the red Duroc pig model. J. Dermatol. Sci..

[162] Wong J.W., Gallant-Behm C., Wiebe C., Mak K., Hart D.A., Larjava H., Hakkinen L. (2009). Wound healing in oral mucosa results in reduced scar formation compared with skin: Evidence from the red Duroc pig model and humans. Wound Repair Regen..

[163] Ghatak S., Khanna S., Roy S., Thirunavukkarasu M., Pradeep S.R., Wulff B.C., El Masry M., Sharma A., Palakurti R., Ghosh N. (2023). Driving adult tissue repair via re-engagement of a pathway required for fetal healing. Mol. Ther..

[164] Yong E., Zhu X., Weng J., Ng M.J.M., Khoo Y.M., Lo Z.J. (2025). Role of therapeutic treatment with antiseptic solutions in the care of diabetic foot ulcers. J. Wound Care.

[165] Seth I., Lim B., Cevik J., Gracias D., Chua M., Kenney P.S., Rozen W.M., Coumo R. (2024). Impact of nutrition on skin wound healing and aesthetic outcomes: A comprehensive narrative review. JPRAS Open.

[166] Attinger C.E., Janis J.E., Steinberg J., Swartz J., Al-Attar A., Couch K. (2006). Clinical approach to wounds: Debridement and wound bed preparation including the use of dressings and wound-healing adjuvants. Plast. Reconstr. Surg..

[167] Barrett S. (2017). Wound-bed preparation: A vital step in the healing process. Br. J. Nurs..

[168] Alam W. (2024). Wound bed preparation and treatment modalities. Clin. Geriatr. Med..

[169] Lin C.-J., Lin C.-W., Wu S.-H., Cheng H.-M. (2025). Effectiveness of maggot debridement therapy for chronic wounds: A systematic review and meta-analysis of randomized controlled trials. Wound Repair Regen..

[170] Salehi S.H., Momeni M., Vahdani M., Moradi M. (2020). Clinical value of debriding enzymes as an adjunct to standard early surgical excision in human burns: A systematic review. J. Burn Care Res..

[171] Schiestl C.M., Moiemem N., Duhamel P., Jones I., Zamparelli M., Lopez-Gutierrez J.C., Kuepper S. (2025). Use of Integra dermal regeneration template bilayer in burn reconstruction: Narrative review, expert opinion, tips and tricks. Eur. Burn J..

[172] Chen J., Wang J., Hart D.A., Ahmed A.S., Ackermann P.W. (2022). Complement factor D as a predictor of Achilles tendon healing and long-term patient outcomes. FASEB J..

[173] Chen J., Wang J., Hart D.A., Zhou Z., Ackermann P.W., Ahmed A.S. (2023). Complement factor D regulates collagen type I expression and fibroblast migration to enhance human tendon repair and healing outcomes. Front. Immunol..

[174] Hart D.A., Ahmed A.S., Chen J., Ackermann P.W. (2024). Optimizing tendon repair and regeneration: How does the in vivo environment shape outcomes following rupture of a tendon such as the Achilles tendon?. Front. Bioeng. Biotechnol..

[175] Medvedeva E.V., Grebenik E.A., Gornostaeva S.N., Telpuhov V.I., Lychagin A.V., Timashev P.S., Chagin A.S. (2018). Repair of damaged articular cartilage: Current approaches and future directions. Int. J. Mol. Sci..

[176] Na Y., Shi Y., Liu W., Jia Y., Kong L., Zhang T., Han C., Ren Y. (2019). Is implantation of autologous chondrocytes superior to microfracture for articular-cartilage defects of the knee? A systematic review of 5-year follow-up data. Int. J. Surg..

[177] Kraeutler M.J., Aliberti G.M., Scilla A.J., McCarty E.C. (2020). Microfracture versus drilling of articular cartilage defects: A systematic review of the basic science evidence. Orthop. J. Sports Med..

[178] Aisenbrey E.A., Tomaschke A.A., Schoonraad S.A., Fischenich K.M., Wahlquist J.A., Randolph M.A., Ferguson V.L., Bryant S.J. (2019). Assessment and prevention of cartilage degeneration surrounding a focal chondral defect in the porcine model. Biochem. Biophys. Res. Commun..

[179] Weimer L., Schmidt L.M., Sengle G., Kruger M., Smith A.M., Brandlin I., Zaucke F. (2025). Localization-dependent variations in articular cartilage ECM: Implications for tissue engineering and cartilage repair. Int. J. Mol. Sci..

[180] Lee M.-S., Lin E.C.-Y., Sivapatham A., Leiferman E.M., Jiao H., Lu Y., Nemke B.W., Leiferman M., Markel M.D., Li W.-J. (2025). Autologous iPSC- and MSC-derived chondrocyte implant for cartilage repair in a miniature pig model. Stem Cell Res. Ther..

[181] Berounsky K., Vackova I., Vistejnova L., Maleckova A., Havrankova J., Klein P., Kolinko Y., Petrenko Y., Prazak S., Hanak F. (2023). Autologous mesenchymal stromal cells immobilized in plasma-based hydrogel for the repair of articular cartilage defects in a large animal model. Physiol. Res..

[182] Ozeki N., Kohno Y., Kushida Y., Watanabe N., Mizuno M., Katano H., Masumoto J., Koga H., Sekiya I. (2021). Sundovial mesenchymal stem cells promote the meniscus repair in a novel pig meniscus injury model. J. Orthop. Res..

[183] Ozeki N., Mizuno M., Yanada S., Okada T., Kubota R., Kushida Y., Furuoka H., Endo K., Katano H., Nakamura K. (2023). Autologous synovial mesenchymal stem cell transplantation suppresses inflammation caused by synovial harvesting and promotes healing in a micro minipig repaired meniscus model. Transplant. Proc..

[184] Zhang S., Wong K.L., Ren X., Teo K.Y.E., Afizah H., Choo A.B.H., Lai R.C., Lim S.K., Hui J.H.P., Toh W.S. (2022). Mesenchymal stem cell exosomes promote functional osteochondral repair in a clinically relevant porcine model. Am. J. Sports Med..

[185] Acosta F.L., Metz L., Adkisson H.D., Liu J., Carruthers-Liebenberg E., Milliman C., Maloney M., Lotz J.C. (2011). Porcine intervertebral disc repair using allogeneic juvenile articular chondrocytes or mesenchymal stem cells. Tissue Eng. Part A.

[186] Barczwska M., Jezierska-Wosniak K., Habich A., Lipinski S., Holak P., Maksymowicz W., Wojtkiewicz J. (2018). Evaluation of regenerative processes in the pig model of intervertebral disc degeneration after transplantation of bone marrow-derived mesenchymal stem cells. Folia Neuropathol..

[187] Canseco J.A., Kojima K., Penvose A.R., Ross J.D., Obokata H., Gomoll A.H., Vacanti C.A. (2012). Effect of ligament marker expression by direct-contact co-culture of mesenchymal stem cells and anterior cruciate ligament cells. Tissue Eng. Part A.

[188] Eylert G., Dolp R., Parousis A., Cheung R., Auger C., Holter M., Lang-Olip I., Reiner V., Kamolz L.-P., Jeschke M. (2021). Skin regeneration is accelerated by a lower dose of multipotent mesenchymal stromal/stem cells0a paradigm change. Stem Cell Res. Ther..

[189] Li P., Cao L., Liu T., Lu K., Ma Y., Wang H. (2025). The effect of adipose-derived stem cell (ADSC)-exos on the healing of autologous skin grafts in miniature pigs. Int. J. Mol. Sci..

[190] Harness E.M., Mohamad-Fauzi N.B., Murray J.D. (2022). MSC therapy in livestock models. Transl. Anim. Sci..

[191] Groenen M.A.M., Archibald A.L., Uenishi H., Tuggle C.K., Takeuchi Y., Rothchild M.F., Rogel-Gaillard C., Park C., Milan D., Megens H.-J. (2012). Analysis of pig genomes provide insight into porcine demography and evolution. Nature.

[192] Warr A., Affara N., Aken B., Beiki H., Bickhart D.M., Billis K., Chow W., Eory L., Finlayson H.A., Flicek P. (2020). An improved pig reference genome sequence to enable pig genetics and genomics research. GigaScience.

[193] Lyu Y., Yang Y.-G., Hu Z. (2026). Recet advances in the construction of humanized animal models and applications in translational medicine. Anim. Models Exp. Med..

[194] Igbal M.A., Hong K., Kim J.H., Choi Y. (2019). Severe combined immunodeficiency pig as an emerging animal model for human disease and regenerative medicines. BMB Rep..

[195] Gun G., Kues W.A. (2014). Current progress of genetically engineered pig models for biomedical research. Biores. Open Access.

[196] Hill A.B., Murphy Y.M., Polkoff K.M., Edwards L., Walker D.M., Moatti A., Greenbaum A., Piedrahita J.A. (2024). A gene edited pig model for studying LGR5+ stem cells: Implications for future applications in tissue regeneration and biomedical research. Front. Genome Ed..

[197] Arias K.D., Fernandez I., Gutierrez J.P., Bozzi R., Alvarez I., Goyache F. (2014). Characterizing local pig breeds as reservoirs for the domestic pig genetic variability worldwide via contributions to gene diversity and allelic richness. J. Anim. Sci..

[198] Li L., Pang D., Wang T., Li Z., Chen L., Zhang M., Song N., Nie D., Chen Z., Lai L. (2009). Productor of a reporter transgenic pig for monitoring Cre recomginase activity. Biochem. Biophys. Res. Commun..

[199] Zhu H.-T., Yu L., Lyu Y., Wang B. (2014). Optimal pig donor selection in islet xenotransplantation: Current status and future perspectives. J. Zhejiang Univ. Sci. B.

[200] Corr D.T., Hart D.A. (2013). Biomechanics of scar tissue and uninjured skin. Adv. Wound Care.

[201] Rananmukhaarachchi S.A., Lehnet S., Ranamukhaarachchi S.L., Sprenger L., Schneider T., Monsoor I., Rai K., Hafeli U.O., Stoeber B. (2016). A micromechanical comparison of human and porcine skin before and after preservation by freezing for medical device development. Sci. Rep..

[202] Germscheid N.M., Thornton G.M., Hart D.A., Hildebrand K.A. (2011). A biomechanical assessment to evaluate breed differences in normal porcine medial collateral ligaments. J. Biomech..

[203] Germscheid N.M., Thorton G.M., Hart D.A., Hildebrand K.A. (2012). Wound healing differences between Yorkshire and red Duroc porcine medial collateral ligament identified by biomechanical assessment of scars. Clin. Biomech..

[204] Fermor H.L., McLure S.W.D., Taylor S.D., Russell S.L., Williams S., Fisher J., Ingham E. (2015). Biological, biochemical and biomechanical characterization of articular cartilage from porcine, bovine and ovine hip and knee. Biomed. Mater. Eng..

[205] Vardaxis N.J., Brans T.A., Boon M.E., Kreis R.W., Marres L.M. (1997). Cofocal laser scanning microscopy of porcine skin: Implications for human wound healing studies. J. Anat..

[206] Debeer S., Le Luduec J.-B., Kaiserlian D., Laurent P., Nicolas J.-F., Dubois B., Kanitakis J. (2013). Comparative histology and immunohistochemistry of porcine versus human skin. Eur. J. Dermatol..

[207] Koopmans S.J., Schuurman T. (2015). Considerations on pig models for appetite, metabolic syndrome and obese type 2 diabetes: From food intake to metabolic disease. Eur. J. Pharmacol..

[208] Schreib C.C., Kelley E.L., Audia G., Gang R., Johnson S., Fleury S., Behun M.N., Vojidova D., Kulkarni M., Donnell G.N. (2026). Cell-based cytokine patch for localized immunomodulation and accelerated healing in rodent and porcine wounds. Nat. Biomed. Eng..

[209] Yang H.-Y., Zhu K., Gallegos A., Recendez C., Villa-Martinez G., Zlobina K., Kesapragada M., Chan S., Hallium J., Alhamo M.A. (2026). Characterization of cutaneous wound healing in swine. JID Innov..

[210] Verma N., Rettenmeier A.W., Schmitz-Spanke S. (2011). Recent advances in the use of Sus Scrofa (pig) as a model system for proteomic studies. Proteomics.

[211] Hara H., Lin Y.J., Zhu X., Tai H.-C., Ezzelarab M., Balamurugan A.N., Bottino R., Houser S.L., Cooper D.K.C. (2008). Safe induction of diabetes by high-dose streptozotocin in pigs. Pancreas.

[212] McCune J.T., Bezold M.G., Davidson J.M., Serezani C.H., Cook R.S., Duvall C.L. (2026). Transcriptomic profiling of diabetic porcine wound healing model identifies key metabolic, inflammatory, and oxidative stress pathways. Wound Repair Regen..

[213] Yang Z., Ren L., He T., Yang P., Liu X., Di C., Wang R., Gap D., Jin L., Li C. (2026). On-demand hydrogen sulfide-releasing hydrogel reprogrammin the diabetic wound microenvironment to accelerate healing. Biomaterials.

[214] Chaudhuri S., Nguyen H., Rangayyan R.M., Walsh S., Frank C.B. (1987). A Fourier domain directional filtering method for analysis of collagen alignment in ligaments. IEEE Trans. Biomed. Eng..

[215] Liu Z.Q., Rangayyan R.M., Frank C.B. (1991). Statistical analysis of collagen alignment in ligaments by scale-space analysis. IEEE Trans. Biomed. Eng..

